# Healthcare Providers’ Satisfaction with Implementation of Telemedicine in Ambulatory Care during COVID-19

**DOI:** 10.3390/healthcare10071169

**Published:** 2022-06-22

**Authors:** Arwa Althumairi, Alaa Fathi AlHabib, Arwa Alumran, Zahraa Alakrawi

**Affiliations:** Department of Health Information Management and Technology, College of Public Health, Imam Abdulrahman Bin Faisal University, Dammam 31441, Saudi Arabia; 2210500064@iau.edu.sa (A.F.A.); aalumran@iau.edu.sa (A.A.); zalakrawi@iau.edu.sa (Z.A.)

**Keywords:** telemedicine, physicians, satisfaction, ambulatory services, COVID-19

## Abstract

Introduction: Telemedicine has become a critical aspect of healthcare provision during the coronavirus pandemic (COVID-19). However, healthcare providers’ utilization of and satisfaction with telemedicine technologies could have a significant impact on the quality of care provided to patients during COVID-19. The current study explores the key factors that could affect healthcare providers’ satisfaction with telemedicine in ambulatory care during the pandemic. Objectives: This research study aims at identifying the factors that could influence the healthcare providers’ satisfaction level with the use of telemedicine in ambulatory care services in Saudi Arabia during COVID 19. Methods: This is a descriptive quantitative cross-sectional study. The research team has utilized the Service Quality Model (SERVQUAL) to assess the healthcare providers’ satisfaction with telemedicine in ambulatory care through a questionnaire that was adapted from previous studies. This questionnaire includes the following dimensions: tangibility, reliability, responsiveness, assurance, and empathy. It was distributed to all ambulatory care physicians in a public hospital-based ambulatory health center in Eastern Region, Saudi Arabia. Results: The study findings showed that Saudis are significantly more satisfied with telemedicine compared to non-Saudis. Age, gender, experience, medical specialty, and computer literacy skills were not found to have any significant effects on the level of the provider’s satisfaction. Conclusion: This research provides new insight and understanding of the relationship between the frequent use of the health information system and the level of physician satisfaction. This major finding puts more emphasis on the importance of education and training when it comes to the adoption of telemedicine through the frequent use of health information systems and applications. These encouraging findings provide a vital piece of information for healthcare organizations interested in a further adoption of telemedicinal practices and applications.

## 1. Introduction

Telemedicinal applications were initially adopted in healthcare to promote access to healthcare services in rural and remote areas and to broaden the scope of healthcare delivery [1]. Telemedicine is a branch of Information Communication Technology that involves the method of communication between healthcare providers and their patients through digital means to decrease personal interaction [2]. This form of communication can be carried out in different forms such as video, audio, and remote patient control devices [3].

However, telemedicine has become a critical aspect of healthcare service provision during the Coronavirus Disease 2019 pandemic (COVID-19). In fact, various telemedicinal applications were used to control and help reduce the spread of COVID-19 [4]. During the pandemic, telemedicine has been used for monitoring patients’ health and reaching the patients at their own homes to avoid the spread of infections [2]. In fact, telemedicine visits have been shown to significantly decrease the risk of COVID-19 spread while providing comparable health outcomes to the patients’ real visits to the outpatient clinics [1]. 

This ability to connect the patients with the healthcare providers regardless of their geographical location has also provided tremendous flexibility with many additional benefits. With this given flexibility, telemedicine has also been shown to reduce absences from work, the cost of travel, and no-show rates in general [1,5]. In fact, Nielsen (2022) has recommended telemedicine as a strategy for continuity and sustainability of care to account for the decline in service utilization post-COVID-19, especially for patients with chronic illnesses [6]. 

Many studies have assessed the adoption of telemedicine during COVID-19 as well as its implementation in ambulatory care [1,2,7]. Telemedicine has been associated with increased levels of satisfaction for both patients and healthcare providers [1,8]. Furthermore, Mason (2021) has concluded that patient satisfaction is critical for the successful implementation of telemedicine. Other studies have also addressed physicians’ satisfactions with telemedicine as well as factors that might influence their adoption such as perceived usefulness and ease of use [3]. 

Healthcare providers’ utilization of telemedicinal technology and applications could have a significant impact on the quality of care provided and patients’ safety. It is also unclear how this form of virtual communication may impact patient engagement and commitment to take care of their health [5,9]. Hence, few studies have addressed the adoption of telemedicine in outpatient settings and physicians’ satisfaction of its implementation. In fact, it is of the utmost importance to address the level of physician satisfaction in order to provide safe channels of communication while maintaining high quality standards of patient care. Thus, this study will explore the factors affecting physicians’ satisfaction with telemedicine and provide evidence-based recommendations as a crucial aspect of its implementation and further adoption in ambulatory care in Saudi Arabia. 

Furthermore, assessing the satisfaction level of physicians with the use of telemedicine in ambulatory services is crucial to providing grounded information to help in identifying and focusing on the major factors that may affect the satisfaction level. This study aimed to focus on the crucial factors that affect the satisfaction level of physicians in the ambulatory services during the COVID-19 pandemic in a governmental hospital-based ambulatory care center in Saudi Arabia. The specific aims were:

1. Assess the satisfaction level of the healthcare providers.
2. Identify factors that impact the satisfaction level when using telemedicine/televisits.

## 2. Material and Methods

### 2.1. Study Design

This study is a descriptive and quantitative cross-sectional study. The outcome variable in this study is the physicians’ satisfaction with using telemedicine. Other independent variables assessed in this study include age, gender, national origin, years of experience, clinical specialty, computer skills, and frequency of using telemedicine/televisits that may affect the satisfaction level. 

### 2.2. Study Population and Setting

This study was conducted in one hospital-based ambulatory care center in the Eastern Region in Saudi Arabia. This ambulatory care center is part of a governmental (public) tertiary-level healthcare system. The name of the study setting will remain anonymous due to data use agreement. The study population includes all the physicians who have been using telemedicine in ambulatory services during COVID-19 and who were working in this public healthcare institution. The survey was distributed from 18 October 2020 to 19 November 2020. The WHO has classified telemedicinal criteria under two aspects: timing of information transmitted, and individual involved. In this study, telemedicine is considered between healthcare provider and patient, with synchronized telemedicine, and any relevant information may be transmitted in a variety of media, such as text, audio, video, or still images [10]. This type of telemedicine was also identified as a televisit [11]. 

The system used in the study hospital to retrieve patient records was MEDiCA CloudCare©, which allows the healthcare provider to enter, retrieve, and monitor patient health records. During the COVID-19 pandemic, the studied hospital provided mobile phones to their healthcare providers to communicate with their patients at a distance. Once the service was completed, they documented their note directly through the patient’s medical record and requested any necessary procedure or services online such as tele-pharmacy and laboratory tests.

### 2.3. Sample Size

A rule of thumb recommended by Comrey and Lee (2013) states that a sample of 100 = fair, 200 = good, 500 = very good, and more than 1000 = excellent [12]. Additionally, using the G Power statical application with a setup estimation of standard error of 0.05 and confidence interval of 95%, the total size needed was 134 [13]. A total of 260 were invited to participate in this research. However, only 132 participants agreed to participate, with a response rate of 50.8%.

### 2.4. Survey Instrument

Physicians’ satisfaction has been assessed using a combination of the Service Quality Model (SERVQUAL) and a survey adapted from three sources in the literature [1,2,3]. An internal consistency for ten participant responses using Cronbach’s alpha test was used to measure the reliability of survey items in scale, and the result was 0.872. A value of more than 0.70 in Cronbach’s alpha is considered reliable [14]. 

### 2.5. Survey Dimensions and Data Items

The satisfaction level was identified based on survey dimensions according to the SERVQUAL model which includes the following dimensions: (1) tangibility; (2) reliability; (3) responsiveness; (4) assurance; (5) empathy [8,14]. The SERVQUAL scale has been broadly used to assess people’s experiences. SERVQUAL items are grouped into five domains: Tangibles; Reliability; Responsiveness; Assurance; Empathy [15], which are described as follows:

1. Tangibles: “physical facilities, equipment, and appearance of personnel” [16]
2. Reliability: “ability to perform the promised service dependably and accurately” [16]
3. Responsiveness: “willingness to help customers and provide prompt service” [16]
4. Assurance “(including competence, courtesy, credibility, and security): knowledge and courtesy of employees and their ability to inspire trust and confidence” [16]
5. Empathy “(including access, communication, and understanding the customer): caring and individualized attention that the firm provides to its customers” [16]

Healthcare service evaluations based on the SERVQUAL scale have been accepted in several countries including UK, Ireland, Spain, Portugal, Turkey, and Korea [15].

A study used SERVQUAL scale to assess an antenatal clinic service and to investigate whether the new service met women’s expectations and to highlight areas needing improvement (Table 1) [15]. Another study used SERVQUAL scale to measure the quality of health care services from the patients’ perspective and to compare the service quality of public and private hospitals in the eastern region of Saudi Arabia [14]. Moreover, a study conducted in Taiwan used SERVQUAL scale to understand the service quality in intensive care units [17]. The survey has a total of 26 data items including demographics and the overall satisfaction score. All questions under each dimension were answered using a 5 point Likert scale [3]. The data collection instrument is provided in the Appendix, Table A1. SERVQUAL is designed to assess the quality of services and users’ satisfaction from the institution to which they belong.

### 2.6. Statistical Analysis

Study data was analyzed using IBM’s Statistical Package for Social Sciences (SPSS) version 26.0 [18]. The physician satisfaction level was treated as a numerical variable and all the other predictors were qualitative (categorical) variables. The physician satisfaction level was treated as a continuous variable and all the other predictors were categorical variables. Mann–Whitney U and Kruskal–Wallis analyses were adopted for bivariate analysis to evaluate differences among study groups at 0.05 level of significance.

The dependent variable was satisfaction score, and independent variables were Nationality, Age, Gender, Specialty, Years of experience, and Computer skills. Before the analysis, the dataset was checked for missing data and outliers. A total of 8 participants had some missing data in the reported scales in some of the domains. The missing data were found to be missing at random; therefore, a multiple imputation was conducted to replace values with average rate. There was a total of 132 participants. Additionally, variance inflation factor (VIF) was assessed to identify multicollinearity that might impact the model stability. An adequate result for multicollinearity is when VIF is >5 as suggested previously in the literature [19].

## 3. Result

Of the 132 participants, 37.9% were between 30 and 39 years old, 38.6% were between 40 and 49 years old, 21.1% were between 50–59 years old, and only 2.3% were >60 years old. Approximately 76% were Saudi and 24% were non-Saudi, and around 66% were males and 34% were females (Table 2). A total of 40% of the participants were from a medical specialty, 53.8% were from a surgical specialty, and only 5.3% were from a dental specialty. The survey study item was normally distributed (as Skewness and Kurtosis were less than 2) illustrated in Table A2 in Apendix Section.

Regarding the years of experience, half of the participants were from 5 to 10 years with 51.5%, 20.2% were from 11 to 15 years, 19.7% were from 16 to 20 years, 13.6% were from 21 to 25 years, and only 3.0% were >30 years of experience. With respect to having good computer skills, 22.7% strongly agreed, 47.7% agreed, 24.2% were neutral, and only 5.3% disagreed. Furthermore, 14.4% strongly agreed that the frequency of using telemedicine/televisits affects telemedicinal satisfaction level, 44.7% agreed, 31.8% were neutral, and 9.1% disagreed.

There were significant differences between SERVQUAL items with *p* value < 0.001. The highest correlations were for Assurance, Tangible, and Responsiveness of Patient/Physicians with scores of r = 0.75, 0.74, and 0.72, respectively (Table 3).

There was a strong significant correlation between survey items and overall satisfaction score (40–70%). Additionally, for the study’s 1st aim, the mean satisfaction score was 55.1 and the standard deviation was 8.4. The continuous variable was normally distributed, and the skewness was 0.002 and kurtosis was −0.542. A value between 2 to −2 is considered normal. Table 1. presents the descriptive statistics of the study participants.

**Figure 1:**
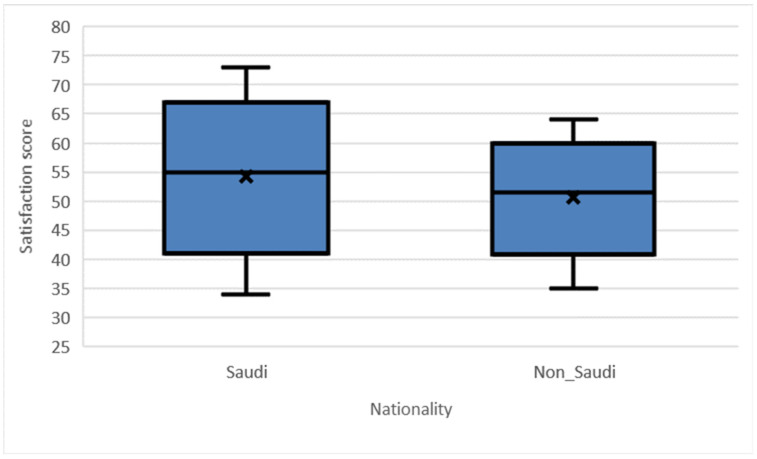
Differences in physician satisfaction score between nationalities, *p*-value = 0.050.

For the study’s 2nd aim, the study results show that Saudi physicians are on average more satisfied with using telemedicine compared to non-Saudi physicians (t = 1231.5, *p* = 0.05, 95% confidence interval between (0.212, 6.956), Table 4). The median satisfaction score for Saudis is 55.0 (IQR: 48.0–61.0), while the median satisfaction score for non- Saudis is 51.5 (IQR: 46.5–56.0), see Figure 1.

**Figure 2:**
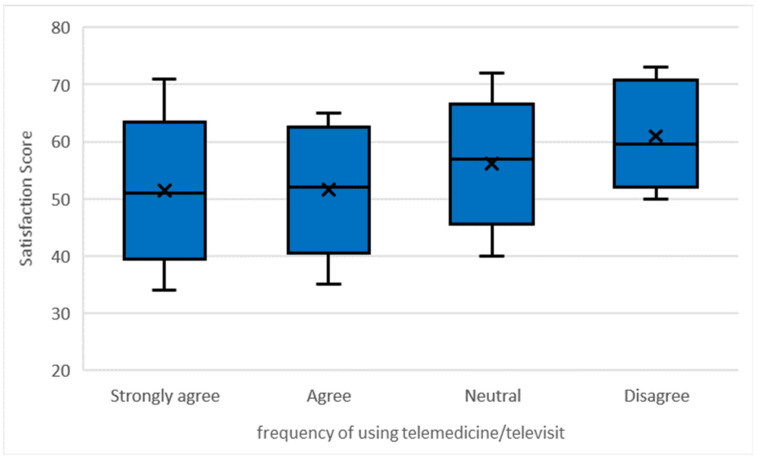
Differences in physician satisfaction score and frequency of using telemedicine/televisit, *p*-value = 0.008.

In addition, there is a statistically significant association between satisfaction level and the frequency of using telemedicine/televisits with *p-* value <0.05 (t = 11.826, *p*-value = 0.008). The median satisfaction score for strongly agree is 51.0 (IQR: 45.0–56.0), agree is 52.0 (IQR:46.0–60.0), neutral is 57.0 (IQR:51.0–61.0), and disagree is 59.5 (IQR:54.0–68.5); see Figure 2.

The other variables show no statistically significant association between themselves and the satisfaction level of the physician, *p-* value > 0.05. Age t = 4.212 (0.240), gender t = 1953.5 (0.985), specialty t= 1.375 (0.502), years of experience t= 2.862 (0.581), and good computer skills t = 2.037 (*p*-value = 0.565) (Table 4).

In addition, a multivariable linear regression was conducted to assess the ‘service quality model’ in the study (Table 4). All of the four dimensions in the study are significantly related to the healthcare provider’s satisfaction score and responsiveness and were the most influential dimension in the model, while empathy was the least influential dimension on the healthcare provider’s satisfaction. Further, 99% of the change in the satisfaction score is attributed to the dimensions in the model (adjusted R^2^ = 0.993, *f* = 3298.195, *p* < 0.0001, Table 5). As the model effect is high, VIF was assessed to exclude multicollinearity. For all dimensions in the model, VIF ranged between 1.098 and 1.487, which indicates no multicollinearity between the dimensions in the model.

## 4. Discussion

This study aimed at assessing the satisfaction level of physicians towards using telemedicine in ambulatory care during the COVID-19 pandemic in Saudi Arabia. The study utilized the SERVQUAL model to achieve this objective, which is considered a major strength of this research. Accordingly, physicians’ satisfaction with telemedicine was assessed through the following dimensions: (1) tangibility, (2) reliability of the system, (3) responsiveness of patients/physicians, (4) empathy, and (5) assurance in addition to the overall satisfaction score. In fact, the SERVQUAL model has been proven to provide be an effective tool for measuring satisfaction as a multi-dimensional construct as suggested by previously in the literature [8,14,20].

This study attempts to identify the factors that could influence physicians’ satisfaction with telemedicine in ambulatory care. All factors including age, gender, nationality, specialty, years of experience, computer skills, and the frequency of using the health information system were tested as potential predictors of the satisfaction level. The results have shown that only nationality and the frequency of using the health information system were found to have statistically significant associations with the satisfaction level. On the other hand, age, gender, specialty, years of experience, and good computer skills did not affect the satisfaction level.

With respect to nationality, Saudi healthcare providers scored higher in overall satisfaction level compared to non-Saudi physicians. In fact, this difference in the satisfaction level proved to be statistically significant. However, to the best of the author’s knowledge, no previous studies have found any evidence suggesting a relationship between physicians’ satisfaction with telemedicine and national origin.

Furthermore, 44.4% of the participants agreed that the frequency of using televisits affected their satisfaction with telemedicine. Since the COVID-19 outbreak, it has been reported that healthcare providers spend more time in the Electronic Health Records (EHRs) systems and applications compared to the pre-pandemic era [7]. We believed that that could possibly be a factor that contributed to healthcare providers’ acceptance of telemedicinal technology. Previous research has suggested that the acceptance of technology is important for the successful implementation of telemedicine in healthcare [21].

These study findings provide a deeper insight into and understanding of the relationship between the frequent use of televisits and the level of physician satisfaction. This means physicians who use televisits on a regular basis have the advantage of more practical and hands-on training and as a result will be more stratified compared to those who do not regularly use televisits (website or application). This eventually can contribute to the faster adoption of telemedicine and its technology and applications [2,22]. Therefore, to streamline the use of telemedicinal services, frequent education, training, and supportive programs must be organized to improve physicians’ satisfaction level and adoption of telemedicine [3,22].

The satisfaction level of physicians and the use of telemedicine was found to be related to the quality of the system used and the system’s acceptance [3]. In addition, to increase physicians’ satisfaction level, telemedicine should be developed to integrate the physicians as well as their patients’ needs [3]. Telemedicine should be well-framed in a way that does not increase physicians workload [3]. Moreover, a previous study showed the importance of the effective use of telemedicine and the need for physicians and healthcare providers to be provided with extensive education on telemedicinal services as this is related to increased satisfaction [1]. Furthermore, physicians who use telemedicinal services should receive adequate training. Findings from previous studies suggest that an unfamiliarity with virtual care solutions is a key barrier to the adoption of telemedicinal services that affects the satisfaction level [2].

Mobile phones were used by the health care providers to communicate with patients during telemedicinal visits. Other studies have used the same method of communication during COVID 19 pandemic in different countries such as Italy [11].

This study has confirmed previous researchers’ findings when it comes to the importance of technological acceptance and training and education providers’ satisfaction with telemedicine. In fact, it is the first study to assess physicians’ satisfaction with telemedicine in Saudi Arabia. Previous research in Saudi has focused more on the current status of electronic health applications during COVID-19 [4] and the use of digital applications during the pandemic [23], and not as much on physicians’ utilization and satisfaction with telemedicine. This is a significant contribution to the body of knowledge and existing research about the Saudi healthcare system.

However, one major limitation for this study is the relatively low sample size, limited to 132 participants, which might limit the metrizability of the study results. In addition, the reliability of the study’s five dimensions survey was verified using Cronbach’s alpha. The result of Cronbach’s alpha shows that all dimensions were reliable with more than 0.7, and this proves that the items for each dimension were coherent and consistent, except that the empathy dimension was less than 0.7 and this might be because of the limited number of items in this dimension. Future research should be invested in identifying more factors that could affect healthcare providers’ acceptance and utilization of televisits. In addition, further research is required to assess healthcare providers’ willingness to use telemedicine in the post-COVID-19 era and the maintenance of telemedicine as part of health information systems’ infrastructure.

## 5. Conclusions

This study is the first study in Saudi Arabia to assess the satisfaction level of physicians for using telemedicine in the ambulatory services during COVID-19 pandemic. The study findings provide a deeper understanding of the relationship between the frequent use of the health information system and the level of physicians’ satisfaction. This encouraging finding provides a vital piece of information for healthcare organizations interested in the further adoption of telemedicinal practices and applications. It will help decision-makers in answering the question of whether the continued use of telemedicine as a platform for service delivery is cost-effective. However, healthcare organizations who would like to continue using telemedicine need to consider additional training requirements for their healthcare providers. The effective use of technology could help promote healthcare organizations, expand telemedicine’s infrastructure to include new services, and increase the level of physician satisfaction in general [22].

During the past months, both healthcare providers and patients have improved their skills and experiences regarding their use of telemedicine [5]. Even after the current significant decrease in the COVID-19 outbreak, telemedicine should be kept as a means of healthcare delivery to sustain control [5]. The decision of healthcare systems to continue to deliver their services through telemedicine, however, depends on the decision of the Ministry of Health. The use of telemedicine during COVID-19 has helped in identifying and analyzing the gaps in the current systems and this will surely help in identifying the success factors in the future [23]. Both healthcare providers and patients are ready to support the expansion of telemedicine [5,9]. They should collaborate to maintain the use of telemedicine in the post-COVID period as it shows significant benefits to patient care [5]. Healthcare providers and patients who witness these benefits must apply pressure to sustain the changes which have helped the safety and quality of patient care [5].

## 6. Clinical Implications

The COVID-19 pandemic has added to the burden of cancer patients. The increased risk of anxiety and depression was clearly demonstrated in this study. Feeling isolated had a greater impact and the anxiety experienced while obtaining COVID-19 information from patient support institutions negatively affected depression. The potential effect of the COVID-19 pandemic can be detrimental to cancer patients. Effective measures should be undertaken to minimize psychological disorders and to provide mental health support. Further related research is needed to identify the extent of the effect of the COVID-19 pandemic on the cognitive, emotional, and psychological distress of cancer patients.

## 7. Study Limitations

The current study had some limitations. Firstly, the limited sample size might have hindered the identification of the relation of some of the factors such as gender. Secondly, the use of a cross-sectional survey-based study limited the generalizability of the study and the ability to assess causation. However, the study used a valid study tool to assess depression and anxiety. Finally, having baseline data on the anxiety and depression levels before the pandemic or/and having a comparison group (patients without cancer) will provide a more accurate comparison. Nevertheless, previous studies support the current study’s findings that people with cancer might be impacted emotionally by any discrepancy in their treatment plan; therefore, the study finding emphasizes the importance of a perfectly monitored treatment plan for patients with cancer.

## Figures and Tables

**Table 1:** Definition of SERVQUAL domains.

SERVQUAL Domains	Operational Definition	Reference	Items Per Domain
Tangibles	Physical facilities, equipment, and appearance of personnel	L. Fan et al., 2017 [16]	4
Reliability	The ability to perform the promised service dependably and accurately	L. Fan et al., 2017 [16]	3
Responsiveness	Willingness to help customers and provide prompt service	L. Fan et al., 2017 [16]	5
Assurance	(Including competence, courtesy, credibility, and security): knowledge and courtesy of employees and their ability to inspire trust and confidence	L. Fan et al., 2017 [16]	3
Empathy	Including access, communication, and understanding of the customer): caring and individualized attention that the firm provides to its customers	L. Fan et al., 2017 [16]	2

**Table 2:** Descriptive statistics of the study participants.

Variables	Frequency	Percentage (%)
**Nationality**		
Saudi	100	75.8
Non-Saudi	32	24.2
**Age**		
30–39	50	37.9
40–49	51	38.6
50–59	28	21.2
>60	3	2.3
**Gender**		
Male	87	65.9
Female	45	34.1
**Specialty**		
Medical	54	40.9
Surgical	71	53.8
Dental	7	5.3
**Years of experience**		
5–10	68	51.5
11–15	26	19.7
16–20	18	13.6
21–25	16	12.1
>30	4	3.0
**Good computer skills**		
Strongly agree	30	22.7
Agree	63	47.7
Neutral	32	24.2
Disagree	7	5.3
**Frequency of using Health Information System affect telemedicine satisfaction level**		
Strongly agree	19	14.4
Agree	59	44.7
Neutral	42	31.8
Disagree	12	9.1
**Satisfaction Score**	Mean (SD): 55.1 (8.4)	

**Table 3:** Pearson correlation score between survey item and overall satisfaction score.

Variables	Mean (SD)	1	2	3	4	5	6
1.Tangible	12.2 (3.1)						
2.Reliability	8.1 (1.7)	0.436 **					
3.Responsiveness	13.7 (3.1)	0.268 **	0.295 **				
4.Empathy	6.3 (1.3)	0.101	0.123	0.171			
5.Assurance	10.1 (2.1)	0.435 **	0.364 **	0.389 **	0.289 **		
6.Satisfaction Score	53.9 (8.4)	0.735 **	0.635 **	0.712 **	0.377 **	0.750 **	

** Correlation is significant at the 0.01 level (2-tailed).

**Table 4:** Significance association between demographic variables and satisfaction score.

Variables	Satisfaction Score			Test Value
	Median	Q1	Q3	(*p*-Value)
**Nationality**				
Saudi	55.0	48.0	61.0	1231.5
Non- Saudi	51.5	46.5	56.0	0.050 *
**Age**				
30–39	57.0	51.0	62.0	4.212
40–49	51.0	47.0	61.0	0.240
50–59	53.0	46.0	58.0	
>60	46.0	46.0	63.0	
**Gender**				
Male	53.0	47.0	61.0	1953.5
Female	54.0	46.0	61.0	0.985
**Specialty**				
Medical	54.0	48.0	62.0	1.375
Surgical	54.0	46.0	61.0	0.503
Dental	52.0	50.0	54.0	
**Years of experience**				
5–10	54.0	47.0	60.0	2.862
11–15	52.5	45.0	62.0	0.581
16–20	52.5	49.0	62.0	
21–25	53.0	47.0	61.0	
>30	59.5	55.0	64.5	
**Good computer skills**				
Strongly agree	54.0	49.0	62.0	2.037
Agree	52.0	46.0	61.0	0.565
Neutral	55.0	51.0	60.0	
Disagree	54.0	48.0	65.0	
**Frequency of using telemedicine/televisit affect satisfaction level**				
Strongly agree	51.0	45.0	56.0	11.826
Agree	52.0	46.0	60.0	0.008 *
Neutral	57.0	51.0	61.0	
Disagree	59.5	54.0	68.5	

* Refers to significant value where *p*-value is <0.05.

**Table 5:** Multivariable linear regression of the survey item.

Survey Item	Beta Standardized Coefficients	Test Score	*p* Value	R^2^	*p* Value
**(Constant)**		−1.548	0.124	0.993	<0.001
**Tangible**	0.385	43.279	<0.001		
**Reliability**	0.229	26.094	<0.001		
**Responsiveness**	0.396	47.350	<0.001		
**Empathy**	0.151	19.332	<0.001		
**Assurance**	0.299	32.813	<0.001		

## Data Availability

The data that support the findings of this study are available from the corresponding author upon reasonable request.

## Appendix A

**Table A1:** Study survey questions.

Demographic Information					
Nationality	Saudi	Non-Saudi			
2. Age	<30	30–39	40–49	50–59	>60
3. Gender	Male	Female			
4. Specialty	Medical	Surgical	Dental		
5. Years of Experience	5–10	11–15	16–20	21–25	>30
6. You have good Computer Skills	Strongly agree	agree	Neutral	Disagree	Strongly disagree
7. What is the method of telemedicine used in your hospital?	Phone calls	Video calls			
8. In your opinion, the Frequency of Using Health Information System affect your telemedicine satisfaction level	Strongly agree	agree	Neutral	Disagree	Strongly disagree
**A.** **Tangibility**					
A sufficient number of computer devices are available in your hospital for use in telemedicine	Strongly agree	agree	Neutral	Disagree	Strongly disagree
2. There is a sufficient IT infrastructure to conduct telemedicine	Strongly agree	agree	Neutral	Disagree	Strongly disagree
3. You have access to a good quality of Internet connection to conduct telemedicine	Strongly agree	agree	Neutral	Disagree	Strongly disagree
4. Security and privacy of information is ensured when using telemedicine	Strongly agree	agree	Neutral	Disagree	Strongly disagree
**B.** **Reliability of System**					
Telemedicine system ease of use	Strongly agree	agree	Neutral	Disagree	Strongly disagree
2. Telemedicine system obstacles	Strongly agree	agree	Neutral	Disagree	Strongly disagree
3. Telemedicine system is user friendly	Strongly agree	agree	Neutral	Disagree	Strongly disagree
**C.** **Responsiveness of Patient/Physicians**					
Patient willingness in using telemedicine	Strongly agree	agree	Neutral	Disagree	Strongly disagree
2. Patient attendance in using telemedicine	Strongly agree	agree	Neutral	Disagree	Strongly disagree
3. Patient acceptance in using telemedicine	Strongly agree	agree	Neutral	Disagree	Strongly disagree
4. Physicians’ acceptance of using telemedicine	Strongly agree	agree	Neutral	Disagree	Strongly disagree
5. Physician’s willingness of using telemedicine	Strongly agree	agree	Neutral	Disagree	Strongly disagree
**D.** **Empathy**					
Telemedicine provides the same treatment outcome	Strongly agree	agree	Neutral	Disagree	Strongly disagree
2. Telemedicine increases physicians’ workload	Strongly agree	agree	Neutral	Disagree	Strongly disagree
**E.** **Assurance**					
There is sufficient ability of the system to conduct e-consultation visits	Strongly agree	agree	Neutral	Disagree	Strongly disagree
2. There is sufficient ability of the system to manage patient’s health status efficiently	Strongly agree	agree	Neutral	Disagree	Strongly disagree
3. You receive enough training using telemedicine	Strongly agree	agree	Neutral	Disagree	Strongly disagree
**Overall Satisfaction with Telemedicine**					
You are satisfied with the telemedicine used in your hospital	Strongly satisfied	satisfied	Neutral	Dissatisfied	Strongly dissatisfied

**Table A2:** Descriptive analysis of the survey item.

Descriptive Statistics									
Study Item	N	Minimum	Maximum	Mean	Std. Deviation	Skewness		Kurtosis	
	Statistic	Statistic	Statistic	Statistic	Statistic	Statistic	Std. Error	Statistic	Std. Error
Tangible	124	6	20	12.20	3.18	0.30	0.22	−0.39	0.43
Reliability	124	3	12	8.06	1.71	−0.12	0.22	0.53	0.43
Responsiveness	124	7	22	13.74	3.14	0.40	0.22	−0.31	0.43
Empathy	124	3	10	6.27	1.26	−0.24	0.22	0.49	0.43
Assurance	124	6	15	10.11	2.12	0.09	0.22	−0.43	0.43
